# Conservation of *Micromeria browiczii* (Lamiaceae), Endemic to Zakynthos Island (Ionian Islands, Greece)

**DOI:** 10.3390/plants10040778

**Published:** 2021-04-15

**Authors:** Anna-Thalassini Valli, Christos Chondrogiannis, George Grammatikopoulos, Gregoris Iatrou, Panayiotis Trigas

**Affiliations:** 1 Laboratory of Systematic Botany, Department of Crop Science, School of Plant Sciences, Agricultural University of Athens, Iera Odos 75, 11855 Athens, Greece; trigas@aua.gr; 2 Laboratory of Plant Physiology, Department of Biology, University of Patras, Rio, 26504 Patras, Greece; fatgiannis@windowslive.com (C.C.); grammati@upatras.gr (G.G.); 3 Laboratory of Botany, Department of Biology, University of Patras, Rio, 26504 Patras, Greece; iatrou@upatras.gr

**Keywords:** monitoring, conservation biology, plant conservation, threatened plant species, population viability analysis

## Abstract

The massive decline in biodiversity due to anthropogenic threats has led to the emergence of conservation as one of the central goals in modern biology. Conservation strategies are urgently needed for addressing the ongoing loss of plant diversity. The Mediterranean basin, and especially the Mediterranean islands, host numerous rare and threatened plants in need of urgent conservation actions. In this study, we assess the current conservation status of *Micromeria browiczii*, a local endemic to Zakynthos Island (Ionian Islands, Greece), and estimate its future risk of extinction by compiling and assessing scientific information on geographical distribution, population dynamics and reproductive biology. The population size and the geographical distribution of the species were monitored for five years. The current population of the species consists of 15 subpopulations. Considerable annual fluctuation of population size was detected. The species is assessed as Endangered according to the International Union for Conservation of Nature threat categories. According to population viability analysis results, its extinction risk was estimated to be 5.6% over the next 50 years, when six of the fifteen subpopulations (40%) might become extinct. The investigation of certain aspects of the species’ biology yielded important data necessary to identify critical aspects for its survival and to propose conservation measures.

## 1. Introduction

According to recent estimates, current plant extinction rates reach up to 1.26 extinctions per year [1]. Plants are an essential component of biodiversity and the foundation for most terrestrial ecosystems. Thus, a decline in plant diversity will be detrimental to all other groups of organisms [2,3] The Mediterranean basin consists an important center for plant diversity, where 10% of the world’s higher plants can be found in an area representing only 1.6% of the Earth’s surface [4]. This biodiversity hotspot has also been the cradle of several of the world’s greatest civilizations, which has resulted in the overexploitation of soil and the conversion of natural habitats into agricultural landscapes [5]. Islands and islets constitute important centers of plant diversity in the Mediterranean [6,7]. Narrow endemism, mainly triggered by geographical isolation and ecological specialization, is a key feature of the Mediterranean island flora, strongly influencing the plant conservation agenda in this region. Island biotas contribute disproportionately to the contemporary extinction crisis [8], and endemic Mediterranean island plants are especially threatened due to habitat loss and fragmentation, climate change, overgrazing and other human-induced activities. Conservation strategies are urgently needed for addressing the ongoing loss of plant diversity, especially the rare and threatened plants of the Mediterranean [9].

The Ionian Archipelago is located in the eastern half of the Mediterranean basin, in western Greece. Τhe recent formation of the Ionian Islands and their close proximity to the mainland have resulted in the establishment of a rich flora principally comprising common species with a low proportion of endemics [10]. Low plant endemism is probably responsible for overlooking the Ionian Islands from plant conservation studies in Greece, and only in recent years has an attempt been made to assess the conservation status of rare and threatened endemic plants of this area [11].

Rare species have an important role in the maintenance of ecosystem functions, because they contribute to the maintenance of the ecosystem diversity, serve as successful indicators of general patterns of species diversity and have a significant impact on invasion resistance, thereby affecting the ecosystem composition and functioning [12,13,14]. The effective conservation of rare plant species requires a detailed understanding of their unique distributions and habitat requirements to identify conservation targets [15]. Ecophysiological traits may have been crucial in the differentiation of narrow endemic species in Mediterranean regions [16]. Photosynthetic traits have been scarcely used in comparing endemic and non-endemic species and have been limited to measuring the maximum photosynthetic rate, A_max_ [17,18]. However, this specific parameter exhibits strong variation even during a single day, as it is strongly influenced by light intensity and comparisons among different species is difficult. On the other hand, the chlorophyll fluorescence parameters, when measured at the dark-adapted state, although not directly related to photosynthetic rates, reflect the fitness of the photosynthetic machinery and the environmental impact on its performance over time [19]. These indices have been used in numerous ecophysiological studies during the last decade, but never in studies comparing the attributes of endemic and non-endemic species.

Understanding the natural history of rare plants is crucial for population management and conservation [20,21,22]. The monitoring of plant populations is one of the core activities of conservation biology. Monitoring can provide critical biological data about rare species [23] and is crucial to identify species that are at risk of extinction [24], assess their conservation status and help improve management decisions [25]. Additionally, such data can lead to the development of effective conservation plans for rare species [26].

Demographic studies can provide information about population trends that is detailed enough to serve as a basis for management decisions [27,28]. Moreover, studies of survival and reproductive patterns are prerequisite to predicting the future growth or decline of populations and to help in the selection of appropriate management strategies for species conservation [29]. An understanding of a rare taxon’s general life history characteristics, reproductive biology, demography and factors constraining population growth is accepted as fundamental for the protection and restoration of the species [23,30,31].

*Micromeria browiczii* Ziel. & Kit Tan is a relatively recently described species [32], endemic to Zakynthos Island. It is a suffruticose perennial, growing on rocky, calcareous, sunny slopes with terra rossa in soil pockets and crevices at 13–416 m above sea level (a.s.l). It is a member of *Micromeria* Benth. sect. *Micromeria*, related to *M. cristata* (Hampe) Griseb., and less closely to *M. cremnophila* Boiss. & Heldr., but it can be distinguished by its neat revolute leaves and dense greyish indumentum [32].

In this study, we assess the current conservation status of *M. browiczii* and estimate its future risk of extinction by compiling and assessing scientific information on geographical distribution, population dynamics and reproductive biology. More specifically, we aim to: (a) define the geographical distribution of the species after exploring all potentially suitable habitats, (b) assess its population dynamics and reproductive biology, and (c) propose strategies and conservation measures for its management and maintenance.

## 2. Results

### 2.1. Geographical Distribution

**Figure 1:**
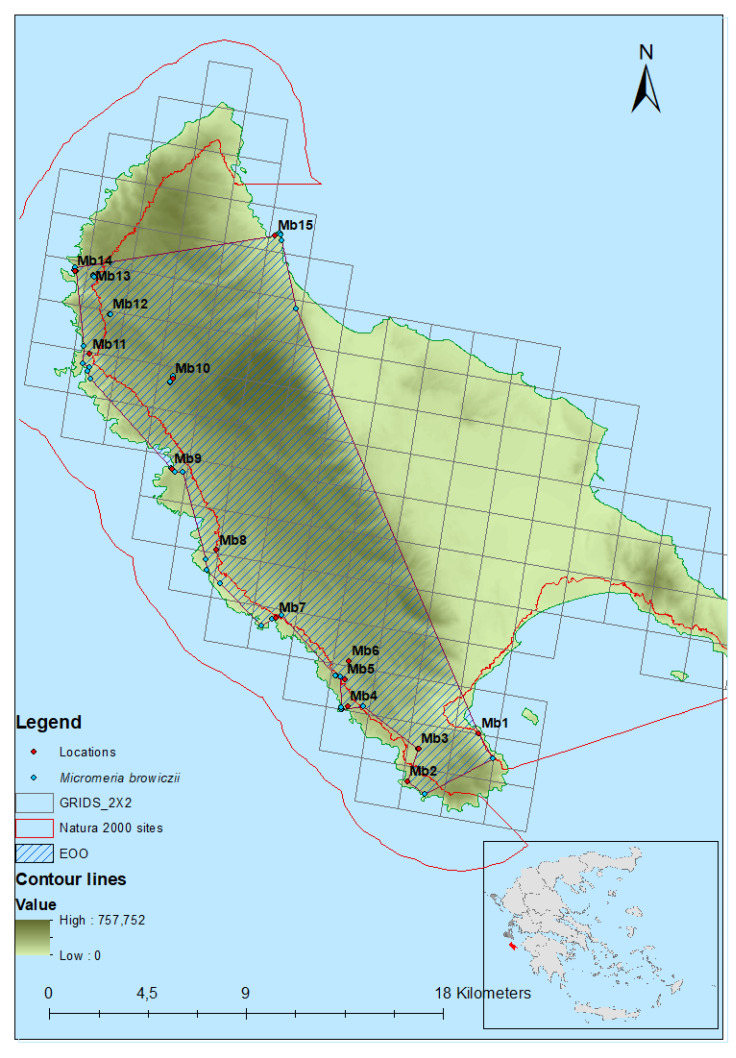
Geographical distribution of *Micromeria browiczii* subpopulations (blue dots) on Zakynthos Island, the estimated extent of occurrence (EOO), and area of occupancy (AOO) based on 2 × 2 km^2^ grid.

The current distributional range of *M. browiczii* is shown in Table 1 and Figure 1. The entire population of the species consists of 15 subpopulations, namely Marathias (Mb1), Faros Keriou (Mb2), Ethniki (Mb3), Plakaki (Mb4), Psaris (Mb5), Agalas (Mb6), Korakonisi (Mb7), Limnionas (Mb8), Kampi (Mb9), Maries (Mb10), Porto Vromi (Mb11), Anafonitria (Mb12), Aghios Georgios (Mb13), Navagio (Mb14), and Xigia (Mb15). During the monitoring period, the species was recorded in 14 new locations, expanding its distributional and altitudinal range. Subpopulations Mb2, Mb4 (colony Pl1), Mb7, Mb8, Mb9 and a part of Mb14 are included within the site of community importance (SCI) “Dytikes kai Voreioanatolikes aktes Zakynthou” (GR2210001), while subpopulation Mb1 is included within the SCI “Kolpos Lagana Zakynthou (Akr. Geraki-Keri) kai nisides Marathonisi kai Pelouzo” (GR2210002) of the Natura 2000 network of protected areas. *M. browizcii* occurs in open rocky (Table 1). According to population monitoring results, the extent of occurrence (EOO) of *M. browiczii* is 195.36 km^2^, the area of occupancy (AOO) based on 2 × 2 km^2^ grid is 68 km^2^, and the local extent of occurrence of all subpopulations is 0.21 km^2^ (Figure 1 and Table 1).

### 2.2. Population Size

*Micromeria browiczii* forms subpopulations and colonies of varying size and extent. Population size (i.e., the total number of mature individuals in all subpopulations), as well as the size of each subpopulation, exhibited considerable annual fluctuations, with the exception of subpopulations Mb6 and Mb12, which exhibited a gradual increase in their size (Table 2). In addition, a reduction in local extent was observed, as the local extent of F1 and Pl2 colonies was dramatically decreased during the monitoring period, due to anthropogenic activities, and more specifically, agriculture and farming (see discussion).

Plant density was higher in 2018, i.e., the year of the smallest local extent of occurrence of all subpopulations (Table 2). The highest plant density was observed in subpopulation Mb12 in 2018. Stage-structure recordings (i.e., number of seedlings, non-reproductive and mature individuals per subpopulation) revealed that all subpopulations were dominated by non-reproductive individuals (61–79%), reproductive individuals represented 9–27%, while the percentage of seedlings was smaller (6–12%) (Table 3).

### 2.3. Reproductive Biology

The mean values of the reproductive characteristics of *M. browiczii* are shown in Table 4. The mean number of flowers per stem and per individual ranged between 3.48 ± 0.36–7.75 ± 0.69 and 22.9 ± 3.7–43.5 ± 8.08, respectively. The mean number of fruits per flower ranged from 1.78 ± 0.17–2.22 ± 0.14. The number of fruits per stem ranged between 28 ± 6.2–46 ± 7.68. The mean number of fruits per individual ranged from 246.2–306.4. Fecundity was highest in 2016, the year with the largest population size. The survival rate of juveniles ranged from 35% in 2017 to 70% in 2018. The mean values of relative reproductive success (RRS, the total percentage of all ovules maturing into seeds) per year were moderate (44.5–55.34%). However, subpopulations or colonies found on old walls showed the greatest values of RRS throughout the monitoring period, while subpopulations growing on limestone cliffs and slopes (which constitute the majority) showed the lowest percentage (Table S1). Pearson’s correlation coefficient revealed a significant positive correlation between RRS and the mean number of fruits per fruiting stem (r = 0.472, *p* < 0.05), as well as with the mean number of fruits per flower (r = 0.9407, *p* < 0.05). Moreover, an irregularity in flowering of mature individuals between successive years was recorded in subpopulation Mb11 during the years 2016–2018. Mature individuals varied tremendously in size, ranging from 8 to 74 cm in diameter and from 3 to 21 cm in height. Furthermore, mature individuals growing in shade conditions exhibited smaller sizes than those growing exposed to full sun. According to our observations, the age of first reproduction of *M. browiczii* is 3 years.

**Figure 2:**
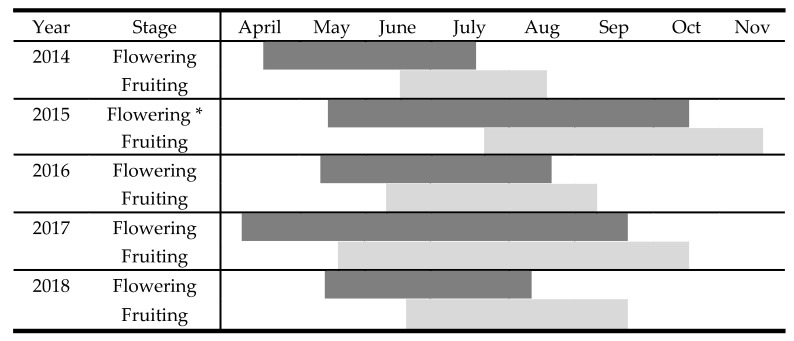
Flowering and fruiting period of *M. browiczii* during five consecutive years (2014–2018). * Subpopulation Mb3 exhibited extended flowering period during 2015 (from 4 May 2015 to 31 January 2016).

The begging and the duration of flowering and fruiting periods of *M. browiczii* exhibited annual fluctuation (Figure 2). Flowering duration was 112 days on average (early May–late August), followed by the fruiting period, which lasts 92 days on average (early June–mid September). The duration of flowering was strongly affected by annual fluctuation in temperature and precipitation (adjR^2^ = 0.836). More specifically, the flowering period was significantly shortened by higher mean annual temperatures (b* = −4.942, *p* < 0.001). Conversely, higher minimum temperatures (b* = 4.113, *p* < 0.001), higher maximum temperatures (b* = 2.745, *p* < 0.001) and increased precipitation (mm) (b* = 0.511, *p* < 0.001) significantly elongated the flowering period. Seedling emergence was observed from November to April.

The insect *Coptocephala rubicunda* subsp. *rubicunda* (Chrysomelidae, Coleoptera) was repeatedly recorded on the flowers of *M. browiczii*. This species is considered to be polinophagous [33], and probably also offers pollination services to *M. browiczii*, resulting in effective pollen transmission between individuals.

### 2.4. Chlorophyll Fluorescence Measurements

**Figure 3:**
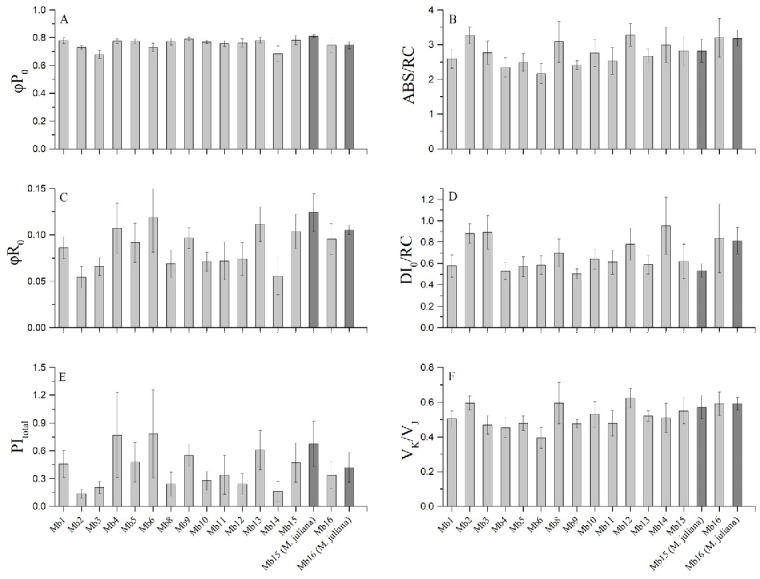
Chlorophyll fluorescence parameters for the different subpopulations of *M. browiczii* (light grey columns) and of *M. juliana* (dark grey columns). Mean values ± SD, (*n* = 10–15). (**A**): Maximum quantum yield of primary PSII photochemistry (φP_0_, A); (**B**): absorbed photon flux per active reaction center (ABS/RC); (**C**): quantum yield for reduction in end electron acceptors at the PSI acceptor side (φR_0_); (**D**): dissipated energy flux per active reaction centre (DI_0_/RC); (**E**): potential for energy conservation from exciton to the reduction in PSI end acceptors (PI_total_) and (**F**): measure of the relative amplitude of K band (V_K_/V_J_) (related to oxygen evolving complex inactivation).

Chlorophyll fluorescence measurements were performed to assess the state and the level of stress on the photosynthetic apparatus of the subpopulations (Figure 3). Maximum quantum yield of primary PSII photochemistry (φP_0_), which is the most conservative index, showed little variation among different subpopulations. The other indices (quantum yield for reduction in end electron acceptors at the PSI acceptor side (φR_0­_), potential for energy conservation from exciton to the reduction in PSI (Photosystem I) end acceptors (PI_total_), absorbed photon flux per active reaction centre (ABS/RC), dissipated energy flux per active reaction centre (DI_0_/RC) and measure of the relative amplitude of K band (V_K_/V_J_)) showed significant variation among subpopulations. The results indicated a major influence of the microenvironment on the performance of the photosynthetic apparatus. A common trend among indices was recorded, with low values of φR_0_ and PI_total_ accompanied by high values of ABS/RC, DI_0_/RC and V_K_/V_J_. More specifically, there was a hindered capacity of electron flow between and around the photosystems (low values of φR_0_ and PI_total_), accompanied by inactivation of reaction centers (high values of ABS/RC), increased heat dissipation (high values of DI_0_/RC) and a looser connection of OEC (Oxygen Evolving Complex) with PSII (high values of V_K_/V_J_).

When comparing the local endemic *M. browiczii* to the widespread *M. juliana* at the same locations, only marginal differences were obtained and a similar profile of energy flow in the photosynthetic apparatus was found (Figure 3). Minor or no significant differences were also recorded between plants growing on different substrates (Figure S1) and between matures and juveniles (Figure S2), while altitude (Figure S3) showed no correlation with any of the fluorescence parameters. Finally, no significant correlation was found between chlorophyll fluorescence parameters and the number of reproductive individuals (Figure S4) or the relative reproductive success (Figure S5).

### 2.5. Population Viability Analysis (PVA)

**Figure 4:**
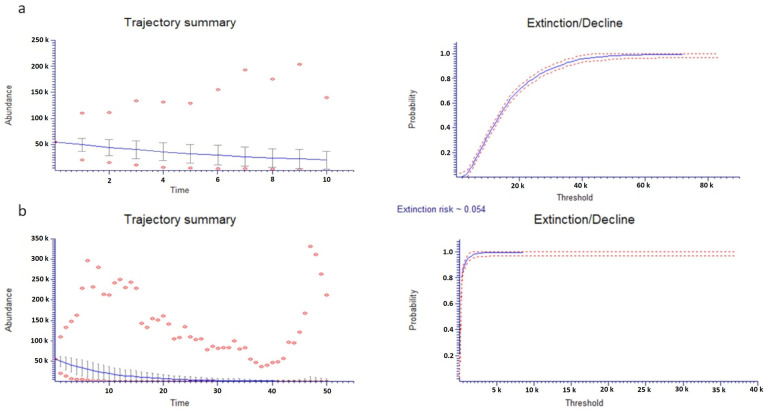
Population viability analysis of *Micromeria browiczii* over (**a**) the next 10 and (**b**) the next 50 years. The average (line), ±1 standard deviation and minimum and maximum (dots) numbers of the population of *M. browiczii* are shown.

PVA in *M. browiczii* using the total number of mature individuals was projected for the next 10 and 50 years (Figure 4). Subpopulations Mb2, Mb3, Mb4, Mb5, Mb8, Mb11 and Mb14 seem to follow a trend of gradual reduction over the next 10 years. However, the population extinction risk is zero. During the next 50 years, species extinction risk increases to 5.6%. There is a high possibility for subpopulations Mb4 (92.1%), Mb5 (91.7%), Mb2 (88.8%), Mb14 (88.3%), Mb3 (73.9%) and Mb11 (66.6%) to go extinct within the next 50 years (Figure S6).

### 2.6. Threats

The direct threats recognized for *M. browiczii* were: (a) threats resulting from agriculture and farming (threat code: 2.3) such as the expansion of agricultural land, resulting in the reduction in local extent at subpopulation Mb4 (colony Pl2); ranching and overgrazing by domestic and semi-domestic animals allowed to roam in the wild, including the impacts of fencing around farmed areas (threat code: 2.3.1) at subpopulations Mb2 (colony F1) and Mb9; (b) agricultural and forestry effluents, such as glyphosate (threat code: 9.3.3) at subpopulation Mb6; (c) tourism and recreational areas (threat code: 1.3) and recreational activities (threat code: 6.1), such as hiking and the construction of secondary roads (threat code: 4.1) at subpopulations Mb5, Mb8, Mb11 and Mb14; gathering of plants (treat code: 5.2) mainly near the hiking trails at subpopulations Mb2, Mb11 and Mb14; housing and urban areas (threat code: 1.1) and specifically villages at subpopulations Mb5, Mb6 and Mb10; and competition with other species (threat code: 12.1) at subpopulation Mb11.

### 2.7. Conservation Status Assessment

Based on monitoring data, *M. browiczii* is classified as Endangered (ΕΝ B1b(iii, iv)c(iv)+2b(iii, iv)c(iv)) [34]. More specifically, the first criterion (B1b(iii, iv)c(iv)) refers to EOO, which was less than 5,000 km^2^, related to the continuing decline estimated for the number of locations or subpopulations, the continuing decline of area–extent–quality of habitat and extreme fluctuations in the number of mature individuals. Criterion B2b(iii, iv)c(iv) refers to AOO, which was less than 500 km^2^, related to the continuing decline estimated for the number of locations or subpopulations, the continuing decline of area–extent–quality of habitat and extreme fluctuations in the number of mature individuals.

## 3. Discussion

Monitoring, including the estimation of species’ geographical range size, population dynamics and exposure to anthropogenic threats, is one of the core activities of conservation biology and provides predictive power [35], as it is essential for determining a species’ conservation status. However, it is time- and resource-consuming, and for these reasons, monitoring programs are especially scarce [36]. When we consider targeting the available conservation resources, island species constitute a rather highly prioritized target, as islands warrant a unique level of attention for biodiversity conservation; they make up only a small percentage of land area but are known for their high endemic species richness [37,38]. In this study, all extant subpopulations of *M. browiczii*, a local endemic of Zakythnos Island, were monitored for five years. The EOO and the AOO were stable during the monitoring period. However, the local extent, which expresses the true natural extent of the species [11], was significantly decreased in subpopulations Mb2 (colony F1) and Mb4 (colony Pl2) during the year 2018, due to anthropogenic activities/pressures related to agriculture and farming (i.e., cultivation of olive trees, fencing for domestic animals and trampling).

Population size, as well as the size of each subpopulation, showed significant fluctuations during the monitoring period (except for subpopulations Mb6 and Mb12 which showed an increasing trend). These fluctuations were partly generated by local habitat changes and/or in threats/pressures (e.g., in subpopulations Mb2, Mb4, Mb8 and Mb11). A reduction in subpopulation sizes at locations Plakaki, Faros Keriou and Agalas were observed as a result of agricultural expansion and intensification. At Plakaki, the cultivation of olive trees and use of agrochemicals led to a reduction in the size and local EOO of Mb4 subpopulation during 2018. In the same year, a large part of subpopulation Mb2 was fenced for animals, resulting in a reduction in subpopulation size because of trampling. Moreover, at locations Mb8 and Mb11, subpopulation size fluctuated in response to interspecific competition. Specifically, in subpopulations located in forest and scrub openings, surrounded by dense vegetation cover, the expansion of tree and shrub species led to the immediate reduction in *M. browiczii* subpopulation size. Zakynthos Island is characterized by dense vegetation cover, limiting endemic plant taxa to specialized habitats with low disturbance and high stress levels [10]. *M. browiczii* often occurs in open, disturbed areas that are usually colonized by more competitive species after a few years.

The high fluctuation observed in the number of mature individuals of *M. browiczii* subpopulations may be also related to climatic conditions (if similar environmental factors affect neighboring subpopulations). The effect of precipitation and mean temperature on the population dynamics of perennial herbs has been demonstrated in several studies (e.g., [39,40]). Understanding their combined effects is a prerequisite for predicting the short-term effects of climate change [41] on population persistence. However, short-time series data may not capture the full range of variation in temperature and precipitation typical for the study area. We would need additional data from longer-term monitoring to be able to confirm the effect of environmental stochasticity/variability on population dynamics.

Seedling survival showed that the transition from juvenile to mature individuals had the same pattern as population size, being lower in 2017. The observed variation in the percent survival of seedlings may be associated with competition. Α correlation between seedling survival and plant density was observed in sampling plots during the monitoring period. Intense inter-specific competition has been associated with a reduction in soil nutrients, water/light availability, and generally a reduction in available resources [42], adversely affecting seedling survival.

Fecundity and relative reproductive success (RRS) were moderate (44.5–55.3%) during the monitoring period, while higher values of RRS were recorded in subpopulations or colonies located in old walls, possibly due to reduced interspecific competition, as fewer coexisting species occur in this substrate. Moreover, walls often provide better moisture conditions than rocks do. Old walls are very porous and the presence of fractures and accumulated sediments common to brick and stone materials increases water storage [43]. Decreased interspecific competition and increased moisture availability possibly explain the increased RRS values observed in walls.

Start date (onset), duration and end date of *M. browiczii* flowering is highly variable. The duration of the flowering period fluctuates in response to variation in climatic factors (i.e., mean annual temperature, minimum annual temperature, maximum annual temperature and precipitation). The interaction between temperature and precipitation has been found to influence flowering time [44]. An extended flowering duration in response to increased precipitation during the preceding months has been documented in several studies in Mediterranean ecosystems [45,46]. Individuals of *M. browiczii* are shallowly rooted and therefore dependent on moisture fluctuations close to the soil surface. The shortened flowering period of *M. browiczii* with warmer mean temperatures is probably the result of reduced longevity of individual flowers.

Irregular flowering between subsequent years (i.e., individuals that are not flowering every single year) was observed in ca. 31.25% of the individuals of subpopulation Mb11 during the years 2016–2018. Individuals in this subpopulation are exposed to shaded conditions for several hours per day, as they are found next to vertical cliffs. The probability that a certain individual will flower in a given year depends on both internal (size and age) and external (e.g., light and temperature) conditions [47]. Plants growing in shaded places are smaller in size and produce fewer flowers than those growing in full light. The flowering behavior of perennial polycarpic plants depends on a complex interaction between resources currently used in reproduction, and the resources stored for future reproduction [48]. Thus, flowering in any particular year cannot be independent of the flowering status in the previous year. Moreover, linear regression analysis revealed a significant correlation between the frequency of flowering and the mean monthly temperature during winter (b* = 0.606, *p* < 0.05), suggesting that the conditions experienced by individuals early in the season significantly affect flowering later in the season.

Based on our chlorophyll fluorescence measurements, the local microenvironment seems to be the major contributing factor to variation in fluorescence indices. This variation could be due to many biotic and abiotic environmental parameters, such as temperature, light exposure, water reserves, soil nitrogen content, and symbiotic associations of roots [49]. Having excluded differences in light exposure and temperature through our sampling procedure, we hypothesize that it is water availability at different locations which influences photosynthetic machinery. Indeed, the photosynthesis of species growing on rocky substrates is strongly affected by Mediterranean drought during the summer [50]. Additionally, reproductive maturity is known to affect photosynthesis [51]; however, our results do not confirm this hypothesis.

Monitoring data collected during this study were used to evaluate the conservation status of *M. browiczii* according to IUCN categories and criteria [34]. On this basis, *M. browiczii* should be categorized as “Endangered” (EN). Population viability analysis (PVA) results indicate that six of the fifteen subpopulations of the species, namely Mb1, Mb3, Mb4, Mb5, Mb11 and Mb14, might go extinct within the next 50 years.

According to field observations and PVA results, subpopulations at Marathias, Ethniki, Plakaki, Psaris, Porto Vromi and Navagio should be prioritized for conservation measures. The control and/or reduction in inorganic fertilizers, pesticides, herbicides, insecticides and other agrochemicals due to intensified agricultural production near subpopulations Mb4, Mb5 and Mb6 is required. Moreover, grazing control to reduce trampling pressure in subpopulation Mb2 is deemed necessary. In addition, informing the local authorities and the community in the vicinity of famous recreation areas (i.e., Navagio and Porto Vromi) where accidental trampling has been observed is needed. Ex situ conservation may also be appropriate, since *M. browiczii* produces orthodox seeds, which can be preserved in a seed bank [52]. Seed banking is a necessary and cost-effective ex situ conservation measure, complementary to in situ conservation of wild plants, and it provides a vital source of material to assist in the ecological restoration of damaged and degraded habitats [53]. Seeds should be collected from all extant subpopulations, if possible, to cover the full range of genetic diversity.

## 4. Materials and Methods

### 4.1. Definitions

The terms mature individual, population, subpopulation, population size, location, EOO and AOO are used according to the definitions established by the IUCN [54]. The local extent of occurrence of each subpopulation (local extent) was calculated according to Andreou et al. [55] as the minimum area occupied by individuals of each subpopulation.

According to the definitions established by the IUCN [34], the term mature individuals is defined as “the individuals known, estimated or inferred to be capable of reproduction”. However, in the case of populations with biased adult or breeding sex ratios and where the population size fluctuates, it is appropriate to use lower estimates of mature individuals [34]. Mature (reproductive) individuals of *M. browiczii* are indistinguishable from juveniles (non-reproductive) ones in size. Juveniles of *M. browiczii* are young individuals with a diameter ranging from 2 to 45 cm (vs. 3–60 cm in matures), and a height ranging from 7 to 26 cm (vs. 7–37 cm in matures). In this study, we define mature individuals as the individuals that are flowering and/or fruiting, as the safest method of identifying mature individuals of *M. browiczii*.

### 4.2. Spatial Distribution

The wider area of the known location, as well as all suitable habitats for *M. browiczii* (rocky, calcareous slopes, not far from the coastal cliffs) were surveyed for five consecutive years (2014–2018), in order to delimit its distribution. A GPS device (eTrex 20, Garmin Ltd., Lenexa, Kansas, USA) was used for the capture of location data in the field. Detailed mapping, the polygon of the EOO for the total population, as well as the polygons of the local extent for each subpopulation of the species (i.e., the minimum area polygon or polygons including all the plant colonies not separated by unsuitable habitat at each location) were constructed with ArcGIS 10.5.1 (ESRI) software. AOO was estimated as the sum of the occupied 2 × 2 km^2^ grid cells.

### 4.3. Population Size

For the estimation of population size, censuses of the mature individuals (those that were flowering and/or fruiting) during the flowering/fruiting period in all subpopulations were carried out for five consecutive years. The number of plants per m^2^, which gives a rough estimate of plant density [56] was calculated for each subpopulation by dividing the number of mature individuals by the local extent. In addition, for the investigation of the stage-structure distribution of the species, plants were classified into three categories: seedlings, non-reproductive plants (juveniles, immatures, senescent and non-flowering) and reproductive plants (flowering/fruiting). Random sample surfaces 5 × 5 m^2^ were placed in Mb4 (*n* = 1). Mb5 (*n* = 5). Mb7 (*n* = 4). Mb8 (*n* = 8). Mb11 (*n* = 20) and Mb15 (*n* = 5) subpopulations for recording individuals per life stage. In the remaining subpopulations, censuses of individuals per life stage were carried out for the whole monitoring period.

### 4.4. Reproductive Biology

Certain aspects of the reproductive biology of *M. browiczii* were studied in all subpopulations during 2016 and in subpopulations Mb4, Mb5, Mb6, Mb7, Mb8, Mb11, Mb12 and Mb15 for five consecutive years, as these subpopulations are representative of the altitudinal range and geological substrates in which the species occurs. Fecundity (expressed as mean number of seeds produced per individual) [57] and relative reproductive success (RRS), were studied by tagging randomly selected mature individuals at the beginning of the flowering season. Moreover, in each plot the position of each mature individual was mapped. All monitoring and experimental procedures were approved by the Hellenic Ministry of Environment and Energy, Directorate of Forest Protection (approval no. MEE/DFP/125613/6014).

The numbers of flowering stems per individual and of flowers per flowering stem and per individual were recorded during each flowering period. The number of nutlets per stem was recorded during each fruiting period. In order to estimate the number of sound seeds per flower and per stem, two stems were collected from tagged individuals during each fruiting period and their seeds observed and evaluated for soundness with a stereoscope (Stemi 305 ZEISS, Oberkochen, Germany). RRS was calculated by dividing the actual production of sound seeds to the potential maximum seed production, based on four seeds per flower. Seed rain was estimated according to Andreou et al. [56] by multiplying the estimated number of seeds per individual by the number of mature individuals in each subpopulation and dividing by the local extent of each subpopulation. The survival rate of mature individuals (Sj), the proportion of juveniles that survive from one breeding season to the next, was studied by tagging randomly selected individuals from sample surfaces during the flowering period and checking their viability in the following flowering period. Reproductive biology data were analyzed by substrate (i.e., limestone, gravelly soil and old walls) to examine if RRS is affected by the depth of the organic horizon (Ao) (limestone: Ao = 1.5 cm, gravelly soil: Ao = 7.9 cm, and walls of old buildings: Ao = 0.7 cm), and consequently from interspecific competition in each position.

The duration of flowering and fruiting of *M. browiczii* was monitored every 1–2 weeks over five consecutive years in all subpopulations. The association of annual climate data (mean temperature, maximum temperature, minimum temperature, and precipitation) and the duration of flowering was examined with stepwise multiple linear regression analysis, to investigate the impact of these variables on flowering period. Meteorological data were obtained from Hellenic National Meteorological Service. Statistical analyses were performed with Statistica 8.0 software (StatSoft Inc., Tula, Oklahoma, USA). Moreover, to identify possible pollinators, all insect species that were associated with *M. browiczii* pollination were photographed, and samples were collected for identification to the lowest possible taxonomic level.

Comparisons of data regarding reproductive biology were performed by repeated measures ANOVA. Differences among pairs of means were checked by Tukey’s Method with Statistica 8.0.

### 4.5. Chlorophyll Fluorescence Measurements

Chlorophyll fluorescence measurements were performed during early summer, in mature individuals of *M. browiczii* at all locations, and of *M. juliana* at the two locations where the two species coexist (Mb15 and Mb16). In subpopulations Mb6 and Mb8, juveniles were also measured, while, in Mb5 and Mb14, measurements were taken on individuals growing on different substrates. For all the measurements, intact, fully developed leaves from plants fully exposed to solar irradiation were used, following the JIP-test protocol [19,58]. A high-time resolution portable fluorometer (HandyPEA, Hansatech Instruments, King’s Lynn, Norfolk, UK) was used. Raw fluorescence data were further analyzed according to JIP-test and six indices calculated: φP0, maximum quantum yield of primary PSII photochemistry; φR0, quantum yield for reduction in end electron acceptors at the PSI acceptor side; PItotal, potential for energy conservation from exciton to the reduction in PSI end acceptors; ABS/RC, absorbed photon flux per active reaction center; DI0/RC, dissipated energy flux per active reaction center; and VK/VJ, related to oxygen evolving complex inactivation.

### 4.6. Population Viability Analysis and Conservation Status Assessment

PVA was carried out using the total number of mature individuals during the first year of monitoring (i.e., 2014) as initial abundance and the survival rate of juveniles as survival rate, taking into consideration demographic stochasticity but without considering any density-dependent parameters. Population growth was calculated as the interannual variation in the total number of mature individuals (Nt/Nt+1). The analysis was implemented with provision for the next 10 and 50 years, with RAMAS Ecolab v.2 software [59].

The assessment of the conservation status of *M. browiczii* was assessed following the guidelines of IUCN Red List Categories and Criteria [34].

### 4.7. Threats

The direct threats that have impacted, are impacting, or may impact the status of *M. browiczii*, as well as the stresses they cause to this species were recorded and classified according to IUCN [54].

## Figures and Tables

**Table 1:** Geographical data of *Micromeria browiczii* subpopulations. Abbreviations as follows: SP, subpopulation; Ao, depth of organic horizon; local extent, the minimum area polygon or polygons including all the plant colonies not separated by unsuitable habitat at each location; AOO, area of occupancy.

Location	IUCN SP	Colony	Altitude (m)	Longitude	Latitude	Aspect (°)	Slope (°)	Substrate	Ao (cm)	Local Extent (km^2^)	AOO (km^2^)
Marathias	Mb1	M1	104–112	20.851298°	37.667198°	22.5–67.5 (NE)	45–90	Rock	1.5	0.0001257	8
		M2	21–316			67.5–112.5 (E)					
Faros Keriou	Mb2	F1	141–188	20.807577°	37.656210°	247–337.5 (W. NW)	45–90	Rock	1.5	0.008016	4
		F2									
		F3									
Ethniki	Mb3		154–159	20.812783°	37.669883°	202.5–247.5 (SW)	80	Rock	1.5	0.000027	4
Plakaki (l.c.)	Mb4	Pl1	81–220	20.777832°	37.689664°	247.5–292.5 (W. S)	45–110	Rock	1.5	0.102	4
		Pl2				202.5–247.5 (SW)					
Psaris	Mb5		183–227	20.769923°	37.698439°	247.5–337.5 (NW. W)	20–50	Rock	1.5	0.01119	4
								Gravel	7.9		
Agalas	Mb6		272–287	20.774257°	37.707252°	202.5–247.5 (SW)	45–90	Wall	0.7	0.00218	4
								Rock	1.5		
Korakonisi	Mb7		32–205	20.739322°	37.722931°	22.5–67.5 (NE)	20–45	Rock	1.5	0.01295	4
Limnionas	Mb8		21–178	20.703129°	37.746664°	157.5–247.5 (SW. S)	30–90	Rock	1.5	0.02941	12
Kampi	Mb9	K1	166–182	20.681802°	37.780336°	337.5–360 (N)	10–50	Rock	1.5	0.002303	4
		K2	158–167			292.5–337.5 (NW)					
		K3	163			292.5–337.5 (NW)					
		K4	164			247.5–292.5 (W)					
Maries	Mb10		343–416	20.678940°	37.819131°	202.5–292.5 (SW. W)	45–90	Rock	1.5	0.000653	4
Porto Vromi	Mb11		13–228	20.631936°	37.829057°	112.5–292.5 (SE. S. SW. W)	45–90	Rock	1.5	0.01549	4
Anafonitria	Mb12		310–317	20.644937°	37.844176°	202.5–247.5 (SW)	90	Wall	0.7	0.0000255	4
Aghios Georgios	Mb13		309–325	20.635926°	37.859460°	205.5–247.5 (SW)	10–90	Gravel	7.9	0.000402	4
Navagio	Mb14		200–229	20.625690°	37.862401°	247.5–292.5 (W)	45	Rock	1.5	0.010378	4
								Gravel	7.9		
Xigia	Mb15		10–38	20.731328°	37.879880°	67.5–112.5 (E)	30–90	Rock	1.5	0.006244	8

**Table 2:** Number of mature individuals (subpopulation size), local extent (the minimum area polygon or polygons including all the plant colonies not separated by unsuitable habitat at each location) and plants per m^2^ for each subpopulation per year. Subpopulation abbreviations as in Table 1. SP: subpopulation.

SP	Colony	2014			2015			2016			2017			2018		
		SP Size	L. extent (m^2^)	Plants/m^2^	SP size	L. extent (m^2^)	Plants/m^2^	SP Size	L. extent (m^2^)	Plants/m^2^	SP Size	L. extent (m^2^)	Plants/m^2^	SP Size	L. extent (m^2^)	Plants/m^2^
**Mb1**	**M1**	4	28.1	0.14	2	28.1	0.07	0	28.1	0	2	28.1	0.07	12	28.1	0.43
	**M2**	1	97.5	0.01	11	97.5	0.113	8	97.5	0.08	4	97.5	0.04	81	97.5	0.83
**Mb2**	**F1**	19	3790	0.003	33	3790	0.009	27	3790	0.007	52	3790	0.014	20	250	0.08
	**F2**	2	727	0.003	3	727	0.004	14	727	0.02	7	727	0.01	4	727	0.006
	**F3**	4	9192	0.0004	6	9192	0.0007	35	9192	0.004	11	9192	0.0012	37	9192	0.004
**Mb3**		13	27	0.48	1	27	0.037	11	27	0.4	11	27	0.4	27	27	1
**Mb4**	**Pl1**	124	92,653	0.0013	32	92,652	0.0003	81	92,652	0.0009	41	92,652	0.0004	99	926,52	0.0001
	**Pl2**	43	9411	0.005	17	9411	0.002	92	9411	0.01	152	9411	0.02	43	120	0.36
**Mb5**		3245	11,192	0.3	1354	11,192	0.12	3562	11,192	0.32	1260	11,192	0.112	1632	11,192	0.146
**Mb6**		61	2180	0.028	63	2180	0.028	86	2180	0.039	87	2180	0.04	276	2180	0.13
**Mb7**		1425	12,950	0.11	1166	12,950	0.09	1036	12,950	0.08	259	12,950	0.019	907	12,950	0.07
**Mb8**		782	29,412	0.026	382	29,412	0.012	2147	29,412	0.07	441	29,412	0.015	3824	29,412	0.13
**Mb9**	**K1**	59	1546	0.04	26	1546	0.017	61	1546	0.04	25	1546	0.016	48	1546	0.03
	**K2**	15	17.5	0.86	10	17.5	0.57	14	17.5	0.8	19	17.5	1.09	14	17.5	0.8
	**K3**	14	475.7	0.03	18	475.7	0.038	25	475.7	0.05	20	475.7	0.04	34	475.7	0.07
	**K4**	6	90.8	0.07	0	90.8	0	1	90.8	0.01	0	90.8	0	5	90.8	0.06
**Mb10**		164	653	0.3	336	653	0.5	372	653	0.56	568	653	0.87	347	653	0.53
**Mb11**		926	15,490	0.06	1431	15,490	0.09	1345	15,490	0.086	168	15,490	0.01	168	15,490	0.01
**Mb12**		86	25.5	3.4	185	25.5	7.25	184	25.5	7.2	239	25.5	9.37	274	25.5	10.6
**Mb13**		23	402	0.06	12	402	0.029	29	402	0.07	5	402	0.01	41	402	0.1
**Mb14**		115	10,378	0.01	65	10,378	0.006	88	10,378	0.008	58	10,378	0.0055	54	10,378	0.0052
**Mb15**		1374	6244	0.22	499	6244	0.08	312	6244	0.05	300	6244	0.048	687	6244	0.11
**TOTAL**		**8547**	**22,2831**		**5641**	**222,831**		**9523**	**22,2831**		**3725**	**222,831**		**8579**	**210** **,** **000**	

**Table 3:** Percentage of seedlings, non-reproductive and mature individuals of *Micromeria browiczii* during monitoring period.

Year	Seedlings (%)	Non-Reproductive Individuals (%)	Mature Individuals (%)
2014	12.01	72.29	15.69
2015	6.46	78.69	14.86
2016	12.35	61.06	26.88
2017	12.18	79.18	8.64
2018	6.43	67.61	25.94

**Table 4:** Characteristics of reproductive biology and fecundity (expressed as mean number of fruits produced per individual) of *M. browiczii* during five consecutive years (2014–2018). *n* = sample size (i.e., number of randomly selected mature individuals or number of stems from tagged individuals).

	2014	*n*	2015	*n*	2016	*n*	2017	*n*	2018	*n*
Stems/individuals ± SE	28.1 ± 5 ^a,^*	60	21.24 ± 5.5 ^a^	60	12.2 ± 1.8 ^b^	115	12.38 ± 1.4 ^a^	60	14.7 ± 1.9 ^a^	60
Flowering or fruiting stems per individual (F) ± SE	5.43 ± 0.7 ^a^	60	7.89 ± 1.49 ^a^	60	6.38 ± 1.58 ^a^	115	5.03 ± 0.57 ^a^	60	6.58 ± 0.97 ^a^	60
Mean number of flowers per stem ± SE	7.18 ± 0.99 ^a^	60	3.48 ± 0.36 ^b^	60	5.38 ± 0.55 ^a^	115	7.75 ± 0.69 ^c^	60	4.88 ± 0.55 ^a^	60
Mean number of flowers per individual ± SE	29.25 ± 6.3 ^a^	60	22.9 ± 3.7 ^a^	60	36.4 ± 10.09 ^a^	115	43.5 ± 8.08 ^a^	60	42.55 ± 9.6 ^a^	60
Mean number of fruits (nutlets) per flower	1.78 ± 0.17 ^a^	120	1.93 ± 0.08 ^a^	120	2.22 ± 0.14 ^a^	230	2.095 ± 0.13 ^a^	120	2.13 ± 0.19 ^a^	120
Mean number of fruits (nutlets) per stem (S) ± SE	50.5 ± 6.8 ^a^	120	38.09 ± 5 ^a^	120	48.03 ± 6.5 ^a^	230	48.95 ± 9.2 ^a^	120	40.89 ± 4.06 ^a^	120
Fecundity (mean number of fruits per individual) (S × F)	274.2		300.5		306.4		246.2		269.06	
Seed rain (seeds/m^2^)	0.065		0.028		0.086		0.04		0,047	
Survival of juveniles (%) (Sj)			64.5		45		35		70	
Relative Reproductive Success (%)	44.5 ± 4.35 ^a^		48.26 ± 2.1 ^a^		55.34 ± 3.5 ^b^		52.4 ± 3.2 ^a^		53.6 ± 3.88 ^a^	

Different letters represent significant differences *(p* < 0.05) among the study years, for each reproductive parameters.

## Data Availability

Data is contained within the article or Supplementary Material.

## Supplementary Materials

The following are available online at https://www.mdpi.com/article/10.3390/plants10040778/s1, Figure S1: Chlorophyll fluorescence parameters of mature plants growing on limestone (grey columns) or gravelly (white striped columns) substrate (Mb5 and Mb14 subpopulations), Figure S2: Chloro-phyll fluorescence parameters of mature plants (grey columns) and juvenile plants (white striped columns) of the subpopulations Mb6 and Mb8, Figure S3: Correlation of chlorophyll fluorescence parameters with the altitude of each subpopulation, Figure S4: Correlation of chlorophyll fluores-cence parameters with the number of mature plats of each subpopulation, Figure S5: Correlation of chlorophyll fluorescence parameters with relative reproductive success (RRS, %) of each sub-population, Figure S6: Population Viability Analysis of Micromeria browiczii subpopulations in the next 50 years.

## Abbreviations

ABS/RC	absorbed photon flux per active reaction centre
A_max_	maximum photosynthetic rate
DI_0_/RC	dissipated energy flux per active reaction centre
PI_total_	potential for energy conservation from exciton to the reduction in PSI end acceptors
V_K_/V_J_	measure of the relative amplitude of K band
φP_0_	maximum quantum yield of primary PSII photochemistry
φR_0_	quantum yield for reduction in end electron acceptors at the PSI acceptor side
EOO	Extent of Occurrence
AOO	Area of Occupancy
Local Extent	local extent of occurrence of each subpopulation, i.e., the minimum area polygon or polygons including all the plant individuals/colonies not separated by unsuitable habitat at each location
SP	subpopulation
