# Supplementary material for: Conservation of Micromeria browiczii (Lamiaceae), Endemic to Zakynthos Island (Ionian Islands, Greece)
Source: Plants (Basel). 2021 Apr 15;10(4):778. doi: 10.3390/plants10040778 (PMC8071367; doi:10.3390/plants10040778)
Supplement: Supplementary file 1 [file plants-10-00778-s001.zip › plants-1154290-supplementary.pdf]

plants

MDPI

Supplementary Material

Anna-Thalassini Valli [1,*], Christos Chondrogiannis [2], George Grammatikopoulos [2], Gregoris Iatrou [3] and Panayiotis Trigas [1]

[1] Laboratory of Systematic Botany, Department of Crop Science, School of Plant Sciences, Agricultural University of Athens, Iera Odos 75, 11855 Athens, Greece; trigas@aua.gr

[2] Laboratory of Plant Physiology, Department of Biology, University of Patras, Rio, 26504 Patras, Greece; grammati@upatras.gr (C.C.); grammati@upatras.gr (G.G.)

[3] Laboratory of Botany, Department of Biology, University of Patras, Rio, 26504 Patras, Greece; iatrou@upatras.gr

* Correspondence: thalassinivalli@yahoo.gr

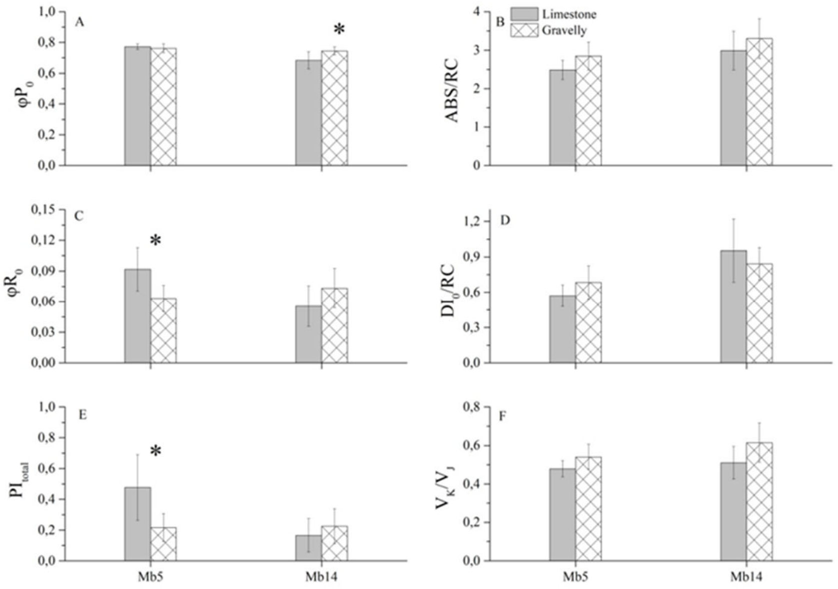

Figure 1. Chlorophyll fluorescence parameters of mature plants growing on limestone (grey columns) or gravelly (white striped columns) substrate (Mb5 and Mb14 subpopulations). Mean values ± SD, (n=10-15). Asterisks indicate statistically significant differences ($p<0.05$) between the different substrates for a given sub-population.

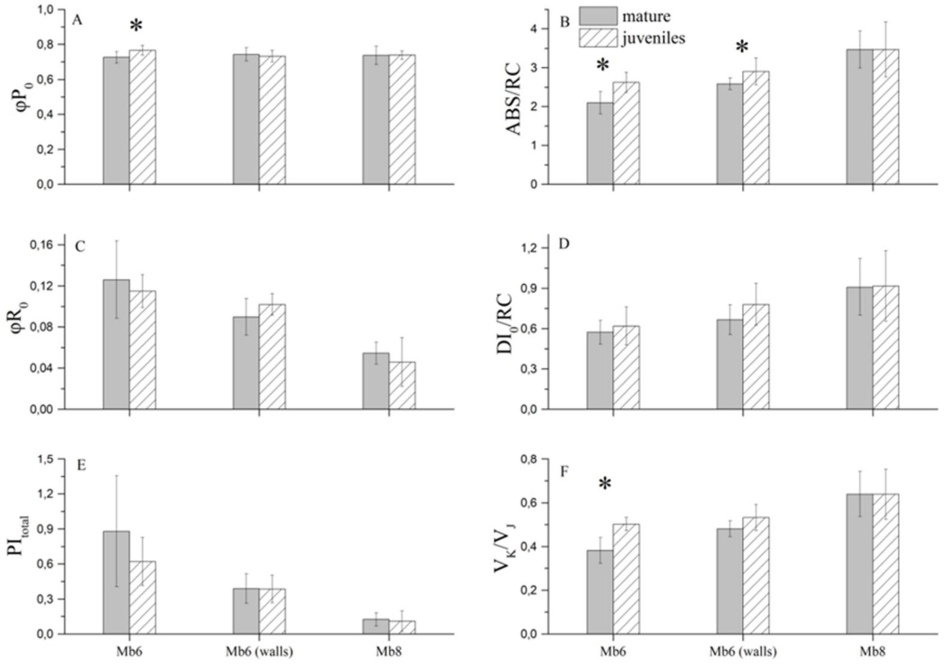

Figure 2. Chlorophyll fluorescence parameters of mature plants (grey columns) and juvenile plants (white striped columns) of the subpopulations Mb6 and Mb8. Mean values ± SD, (n=10-15). Asterisks indicate statistically significant differences ($p<0.05$) between individuals with different reproductive status for a given subpopulation.

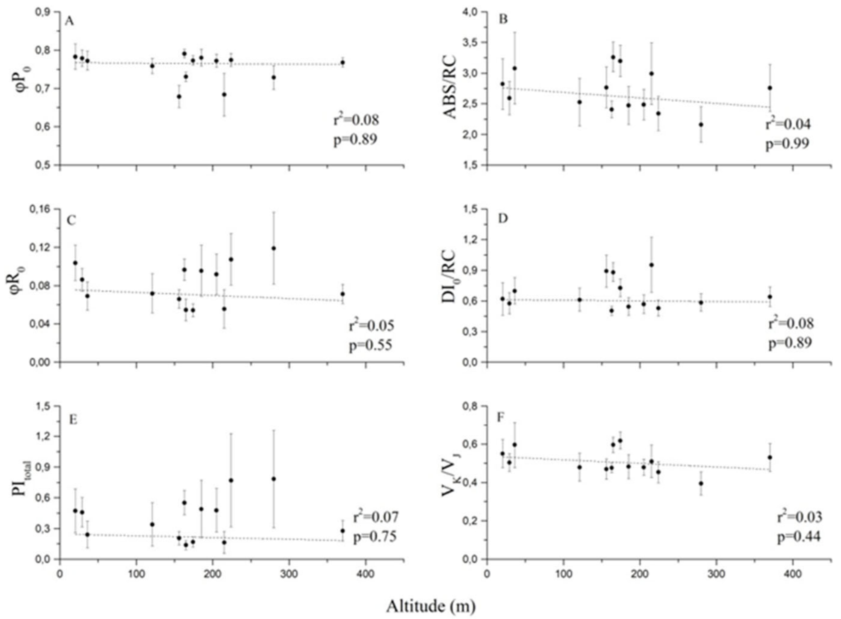

Figure 3. Correlation of chlorophyll fluorescence parameters with the altitude of each subpopulation.

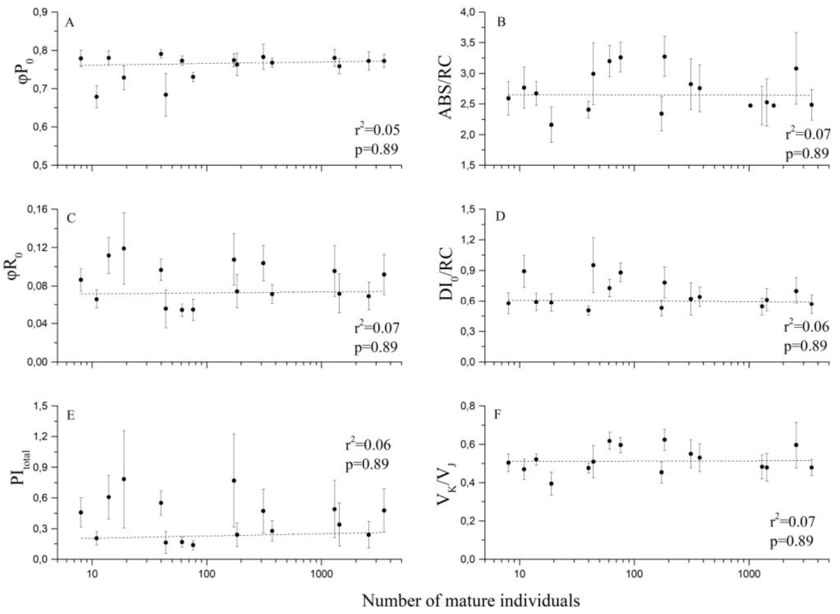

Figure 4. Correlation of chlorophyll fluorescence parameters with the number of mature plats of each subpopulation.

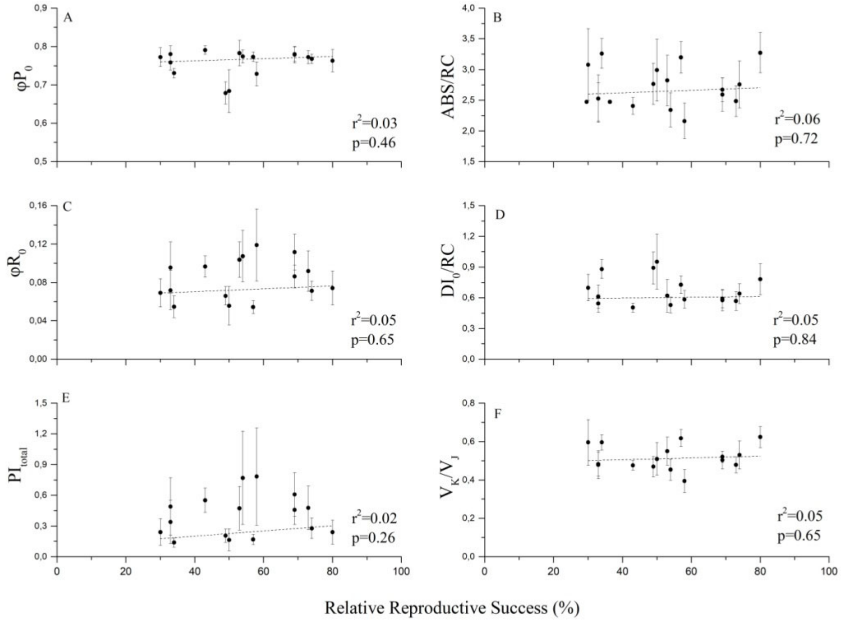

Figure 5. Correlation of chlorophyll fluorescence parameters with relative reproductive success (RRS, %) of each subpopulation.

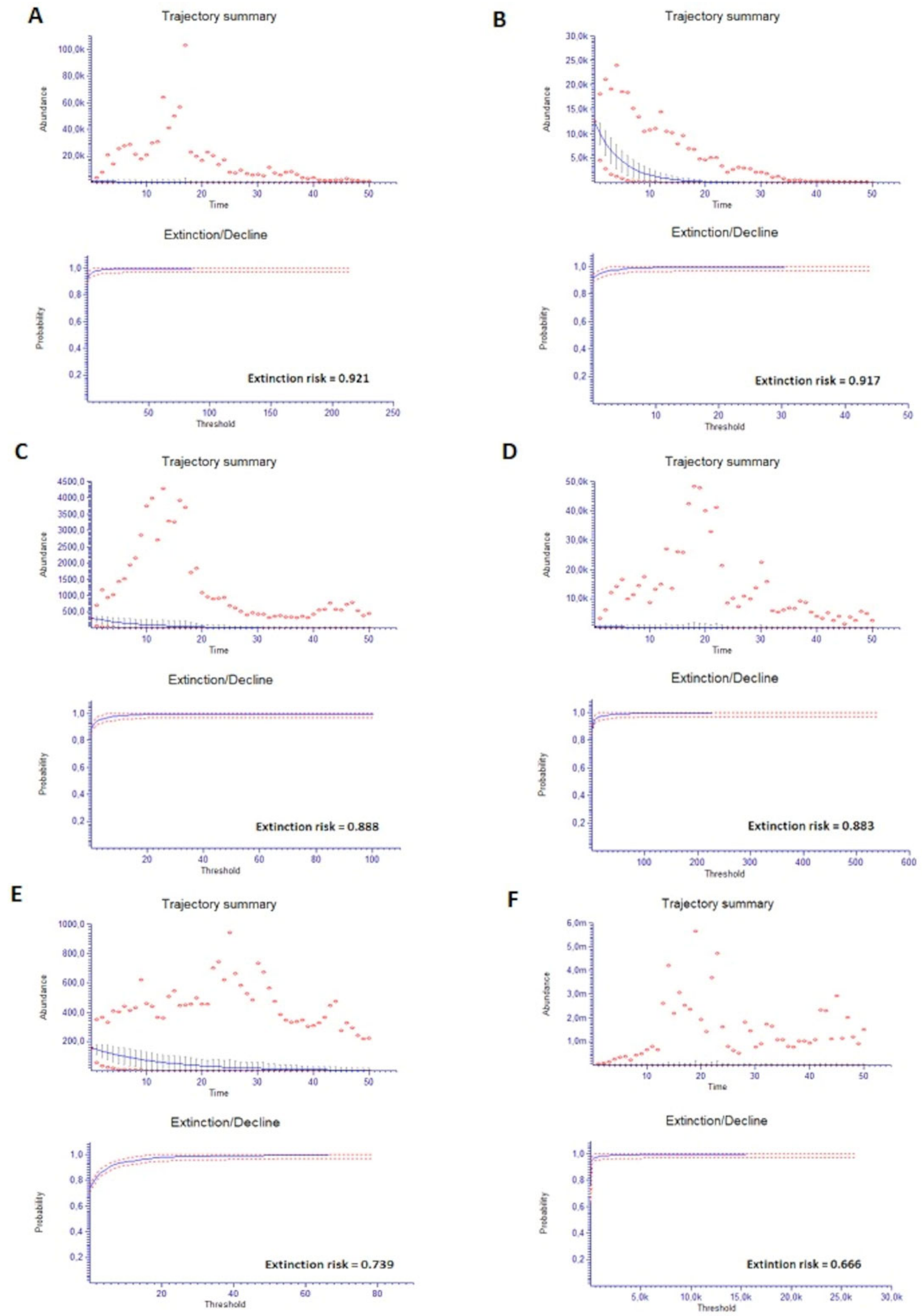

Figure 6. Population Viability Analysis of *Micromeria browiczii* subpopulations in the next 50 years. A) Subpopulation Mb4, B) subpopulation Mb5, C) subpopulation Mb2, D) subpopulation Mb14, E) subpopulation Mb3 and F) subpopulation Mb11. The average (line), ±1 standard deviation and minimum and maximum (dots) numbers of the subpopulations of *M. browiczii* are shown. .
